# Acknowledgment of Reviewers

**DOI:** 10.1002/aps3.1214

**Published:** 2019-01-15

The editors are deeply grateful to the following reviewers (members and nonmembers of the Botanical Society of America) who have generously given of their time to review manuscripts submitted to *Applications in Plant Sciences*. The list includes those who reviewed manuscripts from December 31, 2017, to December 31, 2018. If any reviewer's name has been omitted, we apologize, and ask that a note be sent to the Managing Editor so the list can be corrected.

Allison, Bruce

Amici, Autumn

Arndt, Stefan

Ashley, Mary

Aury, Jean Marc

Aveni‐Deforge, Kyle

Baluska, Frantisek

Barth, Susanne

Bartlett, Megan

Bates, Scott

Bianconi, Andre

Blackmon, Heath

Blischak, Paul

Bueno, C. Guillermo

Campbell, Lesley

Carlson, Craig

Chambers, Sally

Chen, Wei

Chen, Ye

Chica, Eduardo

Cleland, Elsa

Coleman, Craig

Cooper, Laurel

D'Ambrosio, Caterina

D'Odorico, Petra

Davis, Thomas

Dorsey, Brian

Egerton‐Warburton, Louise

Ellwood, Elizabeth

Erickson, David

Esch, Ellen

Estep, Matt

Fang, Zhou

Filippa, Gianluca

Fishbein, Mark

Fonseca, Luiz Henrique M.

Forrest, Laura

Freeland, Joanna

Gabriel y Galan, Jose Maria

Gal, Assaf

Gallaher, Timothy

Garcia de Leon, David

Gardner, Elliot

Gargiulo, Roberta

Ge, Xue‐Jun

Gee, Carole

Gerth, Stefan

Gilbert, Gregory

Grusz, Amanda

Guralnick, Rob

Hamilton, Matthew

Harkess, Alex

Hart, Robbie

Heberling, Mason

Hepler, Nathan

Heyduk, Karolina

Hiiesalu, Inga

Hirsch, Ann

Hodel, Richard

Horner, Harry

Hosseinalizadeh, Mahboubeh

Houben, Andreas

Inouye, David

Jarvis, David

Jellen, Eric

Jinga, Percy

Karabourniotis, George

Kates, Heather Rose

Kenchanmane Raju, Sunil Kumar

Kriechbaumer, Verena

Labonte, Nicholas

Lamb, Eric

Landis, Jacob

Latvis, Maribeth

Leebens‐Mack, Jim

Levy, Richard

Lewis, James

Li, Fay‐Wei

Liu, Luxiang

Livingstone, Kevin

Ljubotina, Megan

Marx, Hannah

Maya‐Lastra, Carlos

McDonnell, Angela

Mei, Wenbin

Meineke, Emily

Merritt, Benjamin

Mithoefer, Axel

Moore, Abigail

Morales‐Briones, Diego F.

Morellato, L. Patricia C.

Mulder, Christa

Munoz‐Concha, Diego

Murrell, Zack

Ng, Wei Lun

Ogutcen, Ezgi

Okumoto, Sakiko

Ozias‐Akins, Peggy

Panchen, Zoe

Park, Daniel

Pasiche‐Lisboa, Carlos

Pau, Stephanie

Pec, Gregory

Pence, Valerie

Pinson, Jerald

Posada‐Perez, Laisyn

Pramod, Sreepriya

Rahaie, Mahdi

Ranathunge, Chathurani

Remington, David

Rocha da Silva, João

Ruhfel, Brad

Sack, Lawren

Santiago, Louis

Savage, Jessica

Schmidt, Marcus

Schneider, Adam

Schori, Melanie

Selvaraj, Michael

Senalik, Christopher

Stevens, Peter

Stieha, Christopher

Stucky, Brian

Sutherland, Brittany

Sweeney, Patrick

Touchell, Darren

Truernit, Elisabeth

Tunison, Robert

Vangansbeke, Pieter

Wäldchen, Jana

Weitemier, Kevin

Williams, Evelyn

Winkler, Daniel

Wyatt, Sarah

Xiao, Yuguo

Yanagi, Tomohiro

Ye, Xingguo

Zenil‐Ferguson, Rosana

Zhao, Peng

Zuntini, Alexandre

Zweck, Justin

